# Reply to: How Many SARS-CoV-2 “Viroporins” Are Really Ion Channels?

**DOI:** 10.1038/s42003-022-03670-9

**Published:** 2022-08-25

**Authors:** Trine L. Toft-Bertelsen, Mads Gravers Jeppesen, Asante Landbrug, Amer Mujezinovic, Bo Hjorth Bentzen, Thomas Nitschke Kledal, Mette Marie Rosenkilde

**Affiliations:** 1 Department of Biomedical Sciences, Faculty of Health and Medical Sciences, University of Copenhagen, Copenhagen, Denmark; 2 Department of Neuroscience, Faculty of Health and Medical Sciences, University of Copenhagen, Copenhagen, Denmark; 3 Synklino ApS, Charlottenlund, Denmark

**Keywords:** Translational research, Target identification

**replying to** N. L. Harrison et al. *Communications Biology* 10.1038/s42003-022-03669-2 (2022)

Viroporins constitute a family of small hydrophobic integral membrane proteins found in RNA and DNA viruses. They vary greatly in terms of structure in their monomers and oligomeric assembly into pores allowing passage of ions. Their functions in virus life cycle and virus-mediated pathology are similarly diverse and span from being central for virus replication, cell entry and egress, and intracellular trafficking to virus particle unpacking and inflammasome activation^1–3^. As integral membrane proteins, in some cases localized to the surface of a virus-infected cell, they are also considered as potential drug targets for future therapeutics against known and emerging viruses. In our recent analyses of transmembrane proteins in SARS-CoV-2^4^, we identified two novel proteins (ORF7b and ORF10) that like the two established viroporins (Protein E and Protein 3a) from this virus could mediate a current upon expression in *X. laevis* oocytes. We moreover identified inhibitors of the activities for all four proteins among known viroporin blockers.

Harrison et al.^5^ express concerns as to whether the reported currents are mediated directly by the viroporins through their functions as ion channels, or whether this current could be caused by indirect effects initiated by release of calcium, and a subsequent activation of calcium sensitive chloride channels. Moreover, concern is raised related to the relatively small current mediated by the four SARS-CoV-2-encoded proteins and their actual expression at the cell surface in the oocytes.

REPLYING TO N. Harrison *Communications Biology* Matters arising 10.1038/s42003-022-03669-2 2022, we appreciate the constructive feedback on our paper^4^, the insightful suggestions and the additional experiments that confirm that amantadine also inhibits the engineered SARS-CoV-2 Protein E. In addition, the presented single channel recordings of SARS-CoV-2 Protein E reconstituted into artificial bilayers are important confirmatory data for ion channel activity of Protein E. We find that the method presented by Cabrera-Garcia et al.^6^ with engineered SARS-CoV-2 Protein E that enhances plasma membrane surface expression is an interesting approach, especially for generating a two-electrode voltage-clamp (TEVC) oocyte assay that can be used for screening drugs against putative viroporins. Hits would obviously need to be confirmed on wild type viroporins as such modifications of a given membrane protein is likely to alter its function and pharmacology. To this end, and to further confirm ion conductance, we have generated inducible cell lines expressing ORF10. Using an automated patch clamp technique with gigaseal formation and Rs compensation (QPatch, Sophion, Denmark), we have in preliminary experiments compared induced vs. non-induced HEK293 cells stably expressing ORF10 under the control of tetracycline. These data suggests that induction of ORF10 expression increases the current amplitude (Fig. 1). This provides a novel and useful platform for future studies of ORF10 in a mammalian expression system and confirm ion channel activity of ORF10 described by us in oocytes^4^.

**Fig. 1:**
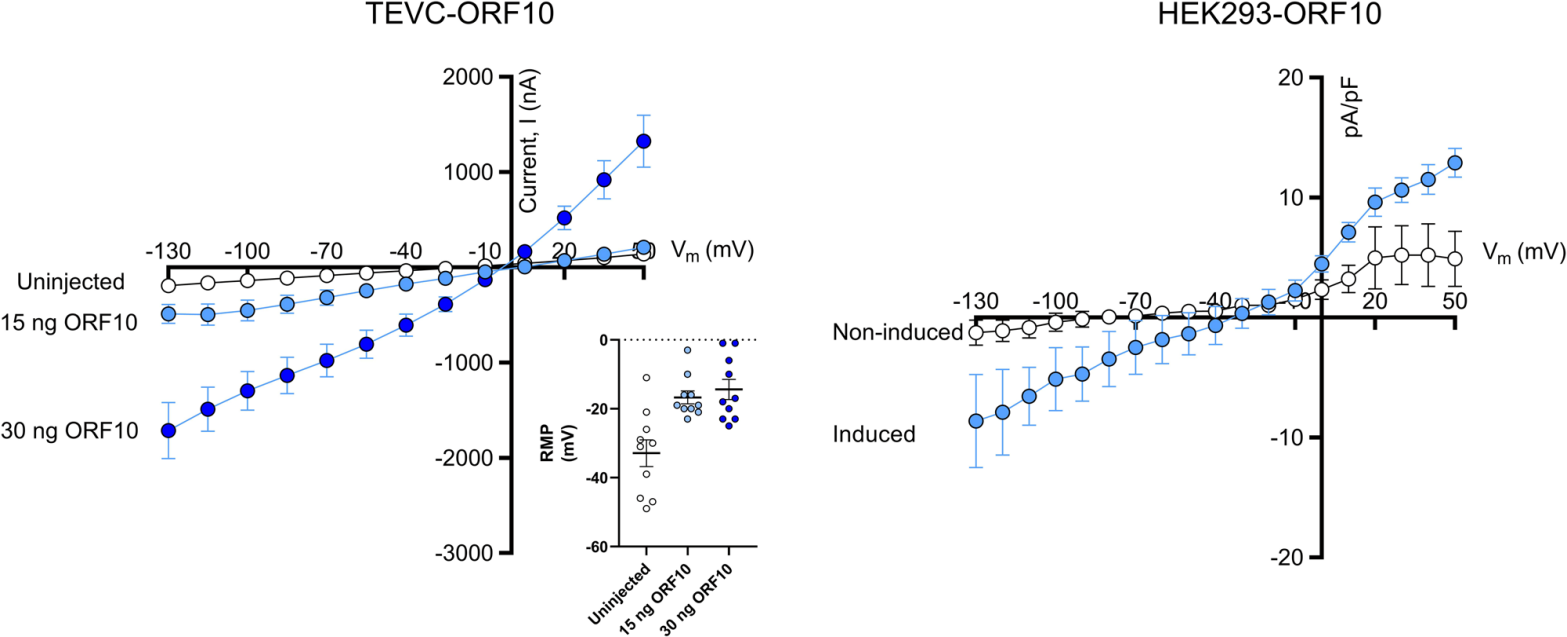
SARS-CoV-2 ORF10 current voltage relationships. (Left) Two-electrode voltage-clamp (TEVC) current-voltage relationships of uninjected (white) *X. laevis* oocytes or oocytes injected with SARS-CoV-2 ORF10, 15 ng (light blue) or 30 ng (blue); oocytes were incubated for 3 days at 19 °C. Data are presented as mean ± SEM, *n* = 10. Inset shows resting membrane potential (RMP). (Right) Automated patch clamp (QPatch) current-voltage relationships of HEK293 cells expressing SARS-CoV-2 ORF10 under tetracycline control, white: non-induced; blue: induced (48 h induction with 500 ng/mL tetracycline). Recordings were performed with physiological solutions. The extracellular solution consisted of (in mM): NaCl 145; KCl 4; CaCl_2_ 2; MgCl_2_ 1; 10 HEPES and 10 glucose. pH = 7.4. The intracellular solution contained (in mM): KCl 120; KOH/EGTA 31.25/10; CaCl_2_ 5.4; MgCl_2_ 1.75; HEPES 10; 4 Na_2_ATP (pH adjusted with KOH to 7.2). Data are presented as mean ± SEM, *n* = 5–6.

We agree that expression of putative viroporins such as Protein 3a, ORF7b and ORF10 in *X. laevis* oocytes also in our hands generate small currents. Hence the contribution by endogenous currents in the oocytes is important to take into consideration. Importantly, we do not observe large currents in uninjected oocytes. Here the average current amounts to −191 ± 37 nA at −130 and 140 ± 16 (*n* = 10) at +50 mV (Fig. 1). From the data provided by Cabrera-Garcia et al.^6^ it is hard to estimate the current sizes in the uninjected oocytes and compare them to ours. Any differences could result from differences in the *X. laevis* oocytes developmental stage, where for instance expression of calcium-activated chloride channels have been reported to change, as also described for other endogenous transport systems in the plasma membranes of oocytes^7^. We use stage 5 oocytes. However, although we thus agree that the current amplitudes of Protein 3a, ORF7b and ORF10 recording in *X. laevis* oocytes are small, we disagree with the suggested signal-to-noise rule stating that only currents with a 30x larger amplitude than the corresponding current recorded in an empty oocyte are trustworthy. Looking back over decades of ion channel research utilizing the two-electrode voltage-clamp technique, currents of around 1–4 μA at +50 mV (current in empty oocyte x 30: 140 nA x 30 = 4.2 µA) are not questioned (e.g., ref. ^8^). Many other factors are important when determining if proteins are ion channels: kinetics, regulation, demonstration of altered function by implementing mutations, selectivity etc.

As presented in our publication, injection of the putative viroporins (Protein 3a, ORF7b and ORF10) resulted in significantly higher current levels as compared to uninjected, albeit still small currents. We thank for pointing out the missing amount of cRNA used in our experiments, which was 20 ng. Preliminary data demonstrates that increasing concentrations of ORF10 results in larger current amplitudes (15 ng measured at −130 mV = −489 ± 100 nA vs. 30 ng −1714 ± 295 nA, mean ± SEM, *n* = 10; see Fig. 1), possible correlated to the number of channels residing in the plasma membrane. In our hands empty oocytes have a resting membrane potential of −33 ± 4 mV. In comparison oocytes injected with SARS-CoV-2 ORF10 15 ng or 30 ng had resting membrane potentials of −17 ± 2 mV and −14 ± 3 mV respectively (Fig. 1, inset). At present we cannot exclude that endogenous calcium-activated chloride channels have an impact on our findings, albeit one would expect currents with significant different amplitudes. Future studies utilizing knock down or pharmacological inhibition of these could help to resolve this issue. However, considering that the viroporins have different pharmacological responses to the various inhibitors tested (see Table 1), we find it unlikely that a common endogenous ion channel (e.g., TMEM16A) is responsible for the observed inhibitory effects amongst the viroporins employed in our study^4^.

**Table 1:** The effects of selected drugs on protein E and protein 3a from SARS-CoV-1 and -2, and ORF7b and ORF10 from SARS-CoV-2.

	SARS-CoV-1		SARS-CoV-2			
	Protein E % inhibition	Protein 3a % inhibition	Protein E % inhibition	Protein 3a % inhibition	ORF7b % inhibition	ORF10 % inhibition
Amantadine	66	ND	77	No effect	No effect	61
Rimantadine	37	ND	No effect	No effect	No effect	No effect
Adamantane	50	ND	No effect	No effect	No effect	No effect
Xanthene	ND	ND	80	20	75	No effect
Emodin	32	26	60	No effect	No effect	No effect
Pyronin B	ND	ND	No effect	No effect	No effect	No effect
Pyronin Y	ND	ND	No effect	No effect	No effect	No effect
HMA	68	ND	58	ND	ND	ND

Summarized effects of 10 µM of amantadine, rimantadine, adamantane, xanthene, emodin, pyronin B, pyronin Y and HMA (hexamethylene-amiloride) on Protein E, Protein 3a, ORF7b and ORF10 expressing oocytes. Inhibitory effects on current activity are indicated as % inhibition. Not determined is indicated as ND. Adapted from ref. ^4^.

We agree that more work will add additional knowledge to the characterization of Protein 3a, ORF7b and ORF10 from SARS-CoV-2 as possible novel ion channels and look forward to presenting more data and read other research groups’ reports. However, our data lay the first corner stone on that journey. Such ion channel activity mediated by viroporins is, as also suggested by Cabrera-Garcia et al.^6^, likely not restricted to plasma cell membrane effects, but could exert its effects in organelles as well, and theoretically these proteins serve more roles than ion conductance.

Future studies such as ion substitution experiments, single channel recording, and mutational analyses to probe for specific channel properties will help to reveal the function of Protein 3a, ORF7b and ORF10, and we welcome such efforts.

## Reporting summary

Further information on research design is available in the Nature Research Reporting Summary linked to this article.

## Supplementary information

Reporting Summary

## Data Availability

The datasets generated during the current study are available from the corresponding author on reasonable request.

## References

[1] To J, Torres J (2018). Beyond channel activity: protein-protein interactions involving viroporins. Subcell. Biochem..

[2] To J, Surya W, Torres J (2016). Targeting the channel activity of viroporins. Adv. Protein Chem. Struct. Biol..

[3] Gargan, S. & Stevenson, N. J. Unravelling the immunomodulatory effects of viral ion channels, towards the treatment of disease. *Viruses* **13**, 2165 (2021). 10.3390/v13112165 PMC8618147 34834972

[4] Toft-Bertelsen TL (2021). Amantadine inhibits known and novel ion channels encoded by SARS-CoV-2 in vitro. Commun. Biol..

[5] Harrison, N. L. A., et al. How many SARS-CoV-2 “Viroporins” are really ion channels? *Commun. Biol.* (2022). 10.1038/s42003-022-03669-2 PMC9411608 36008538

[6] Cabrera-Garcia, D., Bekdash, R., Abbott, G. W., Yazawa, M. & Harrison, N. L. The Envelope protein of SARS-CoV-2 increases intra-Golgi pH and forms a cation channel that is regulated by pH. *J. Physiol.* **599**, 2851–2868 (2021). 10.1113/JP281037 PMC8251088 33709461

[7] Wozniak KL, Phelps WA, Tembo M, Lee MT, Carlson AE (2018). The TMEM16A channel mediates the fast polyspermy block in Xenopus laevis. J. Gen. Physiol..

[8] Schroeder BC, Kubisch C, Stein V, Jentsch TJ (1998). Moderate loss of function of cyclic-AMP-modulated KCNQ2/KCNQ3 K+ channels causes epilepsy. Nature.

