# Publisher Correction: Reply to: How many SARS-CoV-2 “viroporins” are really ion channels?

**DOI:** 10.1038/s42003-022-03982-w

**Published:** 2022-09-27

**Authors:** Trine L. Toft-Bertelsen, Mads Gravers Jeppesen, Asante Landbrug, Amer Mujezinovic, Bo Hjorth Bentzen, Thomas Nitschke Kledal, Mette Marie Rosenkilde

**Affiliations:** 1 Department of Biomedical Sciences, Faculty of Health and Medical Sciences, University of Copenhagen, Copenhagen, Denmark; 2 Department of Neuroscience, Faculty of Health and Medical Sciences, University of Copenhagen, Copenhagen, Denmark; 3 Synklino ApS, Charlottenlund, Denmark

**Keywords:** Translational research, Target identification

Correction to: *Communications Biology* 10.1038/s42003-022-03670-9, published online 25 August 2022.

In the original Article the doi was missing in the sentence: REPLYING TO N. Harrison Communications Biology Matters arising [doi to be added later] 2022.

This has now been corrected to read REPLYING TO N. L. Harrison *Communications Biology* 10.1038/s42003-022-03669-2 (2022) in both the PDF and HTML versions of the Article.

